# Energetic Basis of Microbial Growth and Persistence in Desert Ecosystems

**DOI:** 10.1128/mSystems.00495-19

**Published:** 2020-04-14

**Authors:** Pok Man Leung, Sean K. Bay, Dimitri V. Meier, Eleonora Chiri, Don A. Cowan, Osnat Gillor, Dagmar Woebken, Chris Greening

**Affiliations:** a School of Biological Sciences, Monash University, Clayton, Victoria, Australia; b Department of Microbiology, Biomedicine Discovery Institute, Clayton, Victoria, Australia; c Division of Microbial Ecology, Centre for Microbiology and Environmental Systems Science, University of Vienna, Vienna, Austria; d Centre for Microbial Ecology and Genomics, University of Pretoria, Hatfield, Pretoria, South Africa; e Zuckerberg Institute for Water Research, Blaustein Institutes for Desert Research, Ben Gurion University of the Negev, Sde Boker, Israel; Pacific Northwest National Laboratory

**Keywords:** desert, dormancy, energetics, energy reserve, photosynthesis, trace gas

## Abstract

Microbial life is surprisingly abundant and diverse in global desert ecosystems. In these environments, microorganisms endure a multitude of physicochemical stresses, including low water potential, carbon and nitrogen starvation, and extreme temperatures. In this review, we summarize our current understanding of the energetic mechanisms and trophic dynamics that underpin microbial function in desert ecosystems. Accumulating evidence suggests that dormancy is a common strategy that facilitates microbial survival in response to water and carbon limitation. Whereas photoautotrophs are restricted to specific niches in extreme deserts, metabolically versatile heterotrophs persist even in the hyper-arid topsoils of the Atacama Desert and Antarctica. At least three distinct strategies appear to allow such microorganisms to conserve energy in these oligotrophic environments: degradation of organic energy reserves, rhodopsin- and bacteriochlorophyll-dependent light harvesting, and oxidation of the atmospheric trace gases hydrogen and carbon monoxide. In turn, these principles are relevant for understanding the composition, functionality, and resilience of desert ecosystems, as well as predicting responses to the growing problem of desertification.

## Abstract (precis)

Microbial life is surprisingly abundant and diverse in global desert ecosystems. In these environments, microorganisms endure a multitude of physicochemical stresses, including low water potential, carbon and nitrogen starvation, and extreme temperatures. In this review, we summarize our current understanding of the energetic mechanisms and trophic dynamics that underpin microbial function in desert ecosystems. Accumulating evidence suggests that dormancy is a common strategy that facilitates microbial survival in response to water and carbon limitation.

## INTRODUCTION

**FIG 1:**
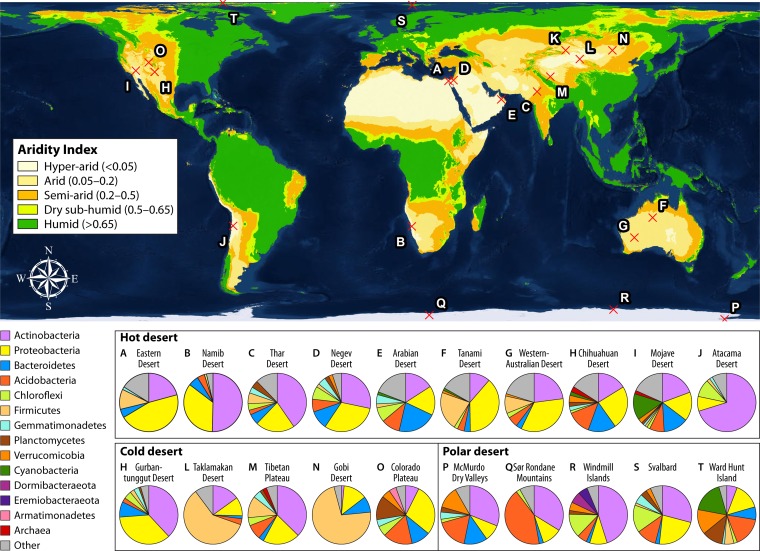
Microbial community structure of global desert soils. The map is generated by ArcGIS 10.6 and shaded by global aridity index, a ratio of mean annual precipitation to potential evapotranspiration (160) modeled by Antonio Trabucco and Robert Zomer (161). The relative abundances of major microbial groups in 20 desert (nonbiocrust) soils from Africa (162, 163), Antarctica (77, 132, 164), Asia (165–170), Australia (171), Europe (172), North America (47, 77, 173), and South America (13) are displayed in pie charts and in Table S1 in the supplemental material. Phyla with a <1% relative abundance were grouped into the category “Other.” *Actinobacteria* is the most abundant phylum detected in bare soils (25.5%), followed by *Proteobacteria* (21%), *Acidobacteria* (6.5%), *Bacteroidetes* (6%), *Chloroflexi* (2.5%), and *Firmicutes* (2%) (median values of the 20 samples are shown in Table S1). Cyanobacteria, though abundant in soil biocrusts and lithic niches, are present in less than 1% in most bare soil samples.

Drylands cover ∼40% of the terrestrial land surface area, with arid and hyper-arid regions constituting 11.5% and 6.4%, respectively (1). Projections based on current global warming trends suggest that drylands will constitute more than half of land surfaces by the end of the century (2). Organisms living in these ecosystems face prolonged and severe water deprivation, which curtail cellular and metabolic activities. Without extracellular water, nutrients and substrates cannot be mobilized in a dissolved form for cellular uptake (3) and microbes themselves are unable to move to find resources, leading to starvation (4). Cellular metabolism is further restricted by environmental stressors, such as low organic carbon and nitrogen availability, high UV irradiation, dryland salinity, and temperature extremes (5–7). In particular, the distribution of key primary producers, such as oxygenic phototrophs, is limited by these cumulative pressures (Fig. 1). Processes such as cyanobacterial photosynthesis in soil biocrusts (8) or water uptake by plant roots (9) generally cease below matrix water potentials of −3 MPa; however, the matrix water potential in desiccated soils is typically between −40 and −95 MPa (10). As a result, in hyper-arid desert soils, photosynthetic organisms are generally restricted to specific lithic refugia, such as the pore spaces of coarse-grained rocks (endoliths) and the ventral surfaces of translucent minerals, such as quartz (hypoliths). Here, they are protected from UV radiation and buffered against extreme temperature and desiccation, while benefiting from sufficient incident light for photosynthesis (11, 12).

10.1128/mSystems.00495-19.1 TABLE S1 Information on published samples of the 20 global desert sites used to construct microbial community profiles. Download Table S1, XLSX file, 0.02 MB. Copyright © 2020 Leung et al. 2020 Leung et al. This content is distributed under the terms of the Creative Commons Attribution 4.0 International license.

Despite photoautotrophs typically being low in abundance, diverse and viable microbial communities are present in the topsoils of most deserts, including the hyper-arid soils of the Atacama Desert (13) and Antarctic Dry Valleys (14). As summarized in Fig. 1, deserts globally are usually dominated by heterotrophic bacteria from phyla such as *Actinobacteria*, *Proteobacteria*, and *Chloroflexi*. In these environments, given that there can be years without precipitation, heterotrophs face extreme starvation for their preferred organic energy and carbon sources. Currently, researchers lack a holistic understanding of the energetic basis of their growth and persistence in desert environments. However, through a combination of culture-based and culture-independent studies, several metabolic mechanisms that may allow these microbes to survive desiccation and associated stresses have been uncovered.

## DORMANCY AS A GENERAL STRATEGY TO REDUCE ENERGY EXPENDITURE

Desert soil microorganisms routinely experience extended periods of desiccation, during which they are subjected to extreme energy limitation and other environmental stresses. In response to these harsh conditions, some microbes reversibly enter a metabolically less active state termed dormancy (15). Different groups of microorganisms may adopt different dormancy strategies. Some species form morphologically distinct resting structures that are commonly characterized by thickened cell walls or accumulations of extracellular polymeric substances (15). For instance, members of *Actinobacteria* and *Firmicutes*, Gram-positive bacterial phyla widely found in drylands (7, 13, 14, 16), are well known for their ability to form highly stress-resistant spores (17, 18). Consistently, these sporulating taxa are among the most common groups identified in desert soils (19–22) based on both conventional cultivation studies and modern molecular phylogenetic analyses. Common *Cyanobacteria* isolated from desert biocrusts, such as *Anabaena*, *Nostoc*, and *Cylindrospermum* (23, 24), can differentiate into specific spore-like structures termed akinetes, which tend to be much larger in size than their vegetative structures (25). Gram-negative *Proteobacteria* isolated from arid soils, such as *Azotobacter* (26) and *Ramlibacter* (27), are likewise able to transit into multilayered cysts. Under favorable conditions, these resting stages can germinate to produce vegetative cells.

A substantial proportion of microorganisms, such as the common arid soil actinobacterium *Arthrobacter* (28), do not undergo extensive morphological differentiation during the transition to the dormant state. However, they still share core strategies with sporulators, including reduction or cessation of growth; reduction of cell size; repression of energetically expensive activities, such as motility and the synthesis of macromolecules; alteration of the composition of membrane lipids and cell wall components; and upregulation of macromolecular repair machinery (15, 29, 30). The highly radiation- and desiccation-resistant genus *Deinococcus* provides an extreme example of how microorganisms can minimize energy expenditure during desiccation persistence. This bacterium tolerates substantial DNA damage, including numerous double-strand breaks (31), without apparently initiating repair in its desiccated state (32). The chromosome is reassembled from the fragments only upon rehydration (33). Accumulation of antioxidants, such as carotenoids, small peptides, and manganese complexes, offers a protective environment for proteins involved in recovery, thereby reducing the energy costs of repair (34, 35). It has been suggested that members of the *Rubrobacteria* (36), a highly abundant desert actinobacterial class (37, 38), adopt similar survival strategies.

Regardless of the form that it takes, dormancy increases cellular resistance to external stresses while reducing energy expenditure. However, dormancy does not completely eliminate the requirement for energy, given that a basal energy supply is required to maintain cellular integrity. Although the exact maintenance energy for microbial communities in soils with low water content has not been reported, modeling studies suggest that microorganisms in moist nutrient-deficient soils may metabolize between 10 and 100 μg of carbon per gram of biomass carbon per hour for maintenance (39), and one experiment demonstrated that desiccated *Arthrobacter* in laboratory conditions consumed 0.0005% of cellular carbon per hour (40). This indicates that dormancy cannot be sustained indefinitely without external energy input. Exacerbating energy demands, hot desert microorganisms are subjected to high levels of oxidative stress due to the high gas permeability of sandy desert soils, desiccation-induced reactive oxygen species formation, Maillard reactions, and extreme temperature-accelerated damage (30, 41–43). The accumulation of excessive damage to nucleic acids, proteins, and cell membranes, if not repaired, will eventually lead to mortality. Therefore, even dormant cells may require basal levels of energy to repair damaged cellular components either during and/or following quiescence. Energy is also required to maintain a minimum membrane potential for ATP synthesis and metabolite transport (44).

## ENERGY RESERVE HYPOTHESIS

In desert ecosystems, a transient water supply can be provided in various ways: occasional precipitation events, condensation of dew or fog, and ice or snow melts in polar deserts. Desert microorganisms may depend on such brief “metabolic windows” to generate biomass and accumulate reserve compounds in preparation for long periods of water scarcity and the consequent requirement of maintenance energy. This constitutes the energy reserve hypothesis, adapted from the “pulse-reserve” paradigm proposed by Noy-Meir in 1973 (45) for plant adaptation in desert ecosystems.

In plant-free desert soils, oxygenic photosynthetic organisms are key primary producers. Such organisms are the keystone taxa of biocrust communities (46, 47) and photosynthetic lithic communities (11, 12, 47). Biocrusts cover up to 70% of semiarid and arid zones on all seven continents (46) and comprise a global area of over 1.3 billion hectares (48). In these environments, water is often provided in the form of early-morning dew, quickly followed by desiccation as the day breaks with rising temperatures and declining relative humidity (49, 50). Microbial communities within the biocrusts must therefore rapidly respond to wetting by resuming respiration and photosynthesis for biomass synthesis and then just as rapidly shut off these systems. For example, simulation of dew hydration in Leptolyngbya ohadii, a dominant cyanobacterium in desert sand biocrusts, causes a rapid resumption of photosynthesis (51). Upon the onset of desiccation, Microcoleus vaginatus, a keystone cyanobacterial species in biocrusts, channels energy into the synthesis of energy and carbon storage compounds, such as polyhydroxyalkanoates, polyphosphates, and cyanophycins (10). Many other cyanobacteria, such as *Scytonema* and *Aphanizomenon*, are also known to accumulate energy reserves in response to water stress or during the transition to dormancy (25, 52, 53).

**FIG 2:**
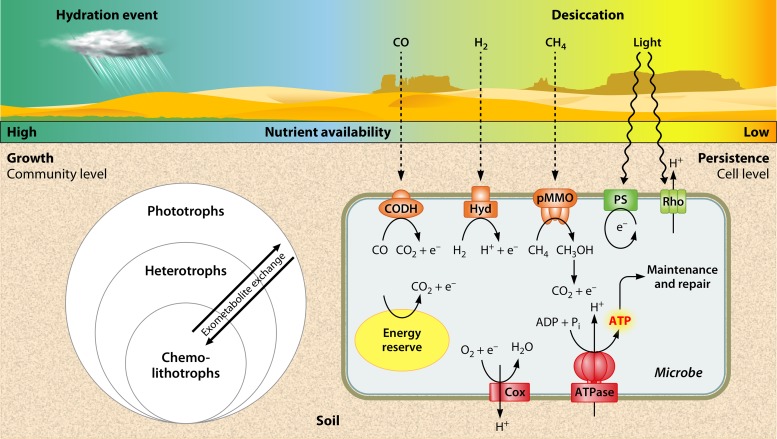
Conceptual diagram representing the model lifestyle of a microbial community in a desert in response to hydration-desiccation cycles. It is proposed that organic carbon reserves (energy reserve hypothesis), light (light-dependent continual-energy-harvesting hypothesis), and trace gases (air-dependent continual-energy-harvesting hypothesis) are the major energy sources that allow dormant microorganisms to persist during prolonged desiccation. Abbreviations: CODH, carbon monoxide dehydrogenase; Hyd, group 1h [NiFe] hydrogenase; pMMO, particulate methane monooxygenase; PS, photosystem of aerobic anoxygenic phototroph; Rho, microbial rhodopsin; and Cox, terminal oxidase.

For heterotrophs, metabolic substrates become available at the instance when soil is wetted. Increases in soil water potential cause the mobilization of extracellular soil organic carbon (54). In addition, due to osmotic changes, hydration is thought to stimulate the release of organic carbon from microorganisms through either inducing cellular lysis (55, 56) or stimulating the secretion of intracellular osmoprotectants, such as trehalose and glycine betaine (57, 58); note, however, that *in situ* evidence for osmolyte release remains lacking (59–61). The sudden availability of metabolizable substrates supports the idea that heterotrophs consume them rapidly, with respiration rates peaking within minutes of soil hydration and then gradually declining (62, 63). This phenomenon is termed the “Birch effect” (64). Upon depletion of these carbon sources, heterotrophs rely on cross-feeding by exometabolite exchange with phototrophs and other heterotrophs (Fig. 2). Cyanobacteria in biocrusts release a large range of photosynthates and exudates, such as hexose sugars, while heterotrophs excrete a smaller subset of metabolites (65, 66). Additionally, biocrust microorganisms, most prominently M. vaginatus, produce large amounts of complex extracellular polymeric substances, such as polysaccharides (67–69), which can potentially be digested by associated specialized heterotrophs like *Bacteroidetes* (70), as observed, for example, in marine consortia (70, 71). The sharing and differential partitioning of this exometabolite pool allows the rapid buildup and accumulation of biomass. In response to xeric stress, heterotrophs upregulate the synthesis of reserve molecules, such as glycogen (72), wax esters (73), and lipids (74).

Chemolithoautotrophs also benefit from trophic interactions with phototrophs and heterotrophs. Diazotrophic *Cyanobacteria* fix nitrogen to ammonia, a portion of which is excreted or leaked from cells (6, 75, 76). This supply of fixed nitrogen supports nitrifying microorganisms, such as *Thaumarchaeota*, which are typically the dominant archaea in desert soils (77–79). In addition, the anaerobic conditions resulting from rapid respiration by heterotrophs after a wetting event can create microenvironments for chemolithoautotrophic anaerobes to flourish (80, 81). This is exemplified by the detection of methanogens in arid soils containing biocrusts, albeit in low abundance (82). Methanogenesis can also be activated by wetting of arid soils, whereby acetoclastic/hydrogenotrophic *Methanosarcina* and hydrogenotrophic *Methanocella* organisms consume fermentative end products produced by heterotrophs as substrates for methane production (78, 83, 84). Chemolithoautotrophs are also known to accumulate energy reserves when under stressed conditions (85–89).

While the energy reserve hypothesis provides a feasible mechanism for maintaining microbial cell energy requirements in desert soils, it is not without limitations. Some deserts may receive insufficient water input over long periods to support this mechanism. Hyper-arid regions in the central Atacama Desert can receive less than 5 mm of rainfall per year (90) and may experience decades without precipitation (6, 13). Likewise, annual precipitation of less than 10 mm in certain areas of the Antarctic McMurdo Dry Valleys is common (91), and a significant fraction of this moisture sublimates (5). The combined effects of other environmental factors may reduce the capacity to generate sufficient energy during one short “water pulse.” For instance, salt accumulated in hyper-arid soils reduces water bioavailability (92–94), the low mean temperatures in Antarctic soils reduce cellular metabolism (95), and the highly limited organic carbon and bioavailable nitrogen in hyper-arid soils may restrict heterotrophic processes (5). Overall, the ability of xerotolerant microorganisms inhabiting desert soils to accumulate and utilize long-term energy storage compounds requires more extensive study, especially *in situ*.

## CONTINUAL-ENERGY-HARVESTING HYPOTHESIS

It is increasingly realized that heterotrophic microorganisms in desert environments possess hidden metabolic flexibility. As elaborated below, they may meet energy demands during starvation by continually harvesting atmospheric trace gases (lithoheterotrophy) or sunlight (photoheterotrophy) as alternative energy sources. These mechanisms are likely to be particularly important in the bare soils of deserts, which are typically dominated by heterotrophic bacterial taxa (Fig. 1), with relatively low numbers of photoautotrophs, such as *Cyanobacteria* (13, 47, 96).

### Light-dependent energy harvesting (photoheterotrophy).

Oxygenic photosynthesis is limited by the availability of its electron donor: water. Water limitation, together with damage of photosystems due to desiccation-induced reactive oxygen species (42) and salt stress (97), is thought to primarily limit the abundance of *Cyanobacteria* in hyper-arid soils. However, it is possible for heterotrophs to derive energy from light by using photons to generate a membrane potential independently of the photosynthetic dark reactions. Two variants of such a light-harvesting mechanism that are dependent on bacteriochlorophyll or rhodopsin (Rho) have been identified (98) (Fig. 2). These processes have sometimes been referred to as “aerobic anoxygenic phototrophy.”

Bacteriochlorophyll (BChl)-dependent light harvesting has been observed in four bacterial phyla: *Proteobacteria* (99), *Chloroflexi* (100), *Acidobacteria* (101), and *Gemmatimonadetes* (102). Unlike *Cyanobacteria*, these bacteria contain only one photosystem, with bacteriochlorophyll *a* being the major photosynthetic pigment (103). The electron transport chain in BChl-dependent light harvesting can operate in a cyclic fashion without exogenous electron donors (103), and therefore, energy can be generated continually with solar input. While BChl-dependent light harvesting is a widespread mechanism for harvesting supplemental energy, particularly in bacteria inhabiting oligotrophic aquatic environments (104, 105), its ecological role in arid soils has not been explored. A culture-dependent study confirmed the presence of soil crust bacterial strains capable of BChl-dependent light harvesting (106). BChl-dependent light harvesting may also be important in hyper-arid Antarctic desert soils. Amplicon sequence screening for genetic determinants of BChl-dependent light harvesting in oligotrophic soils from the Sør Rondane Mountains identified diverse bacteria with this capacity, which were affiliated primarily with the class *Alphaproteobacteria* (107, 108). A subsequent isolation campaign recovered nearly 1,000 isolates, many affiliated with known *Alphaproteobacteria*, harboring BChl-dependent light-harvesting capacity (22). Likewise, a *Hymenobacter* strain (phylum *Bacteriodetes*) was found to have BChl-dependent light-harvesting potential, a trait not previously observed in members from this phylum (22).

Rhodopsin-based light harvesting (Rho-light harvesting) is a minimalistic light energy-harvesting mechanism consisting of a single ion-pumping protein (type I opsin) with a retinal chromophore cofactor (109). This process generates an ion-motive force for ATP synthesis (but not reducing power), potentially providing a survival advantage for microorganisms during nutrient deprivation (110–112). The minimal genetic determinants of this process, namely, a single opsin gene and another gene for retinal synthesis from carotenoid (109), facilitate horizontal gene transfer (113, 114); this has likely enabled the dissemination and diversification of microbial rhodopsins across archaea and bacteria. A global metagenomic survey focused on marine environments estimated that rhodopsin genes are carried by half of prokaryotic taxa and are 3-fold-more abundant than genes for photochemical reaction centers (115). Despite the possible importance of this physiology as a survival strategy in dry oligotrophic habitats, the ecological relevance of Rho-light harvesting in arid soils has received little attention. Analyses of soil crust metagenomes by Finkel et al. indicated that up to half of microbial genomes encode rhodopsins (115). Several more recent studies have focused on Antarctic deserts: while PCR amplification failed to detect rhodopsin genes in soils from the Sør Rondane Mountains (107, 108), a metagenomic study of hypolithic communities from the Miers Valley (McMurdo Dry Valley region) indicated that 20% of bacterial taxa harbored rhodopsin genes (116).

### Atmospheric trace gas oxidation (lithoheterotrophy).

While light-dependent energy harvesting strategies are clearly important physiological processes in desert soil habitats (5, 6, 11, 46), such processes are always constrained by light penetration. Atmospheric trace gases may provide a viable alternative energy source for desert soil microorganisms residing within and below the photic zone. Trace gases, such as hydrogen (H_2_), carbon monoxide (CO), and methane (CH_4_), are ubiquitous, diffusive, and high-energy electron donors. The porosity of dry desert soils, due to their coarse texture and low water retention, may also facilitate trace gas permeation (117). The possibility that these substrates support respiration in desert soil microbial communities should therefore be considered (Fig. 2).

Dihydrogen, as the most fundamental molecule, can serve as an energy source for microorganisms from a wide range of taxa and ecosystems (118, 119). Soil microorganisms scavenge H_2_, which is present at atmospheric mixing ratios of 530 ppbv (120), as an electron donor for aerobic respiration (121). While this process was inferred some four decades ago, the organisms and enzymes responsible for this process have only recently been characterized (122, 123). Genetic and biochemical studies have shown that this process is catalyzed by the group 1h [NiFe]-hydrogenases linked to the respiratory chain; synthesis of this enzyme is induced during nutrient starvation and is critical for long-term survival (124–128). It is now established that atmospheric H_2_ oxidation is a broadly distributed trait among major soil microbial phyla, having been experimentally verified in *Actinobacteria* (126, 128–130), *Acidobacteria* (125, 131), and *Chloroflexi* (124). The genetic determinants of this activity were found to be carried by at least five additional cultured microbial phyla (123) and two candidate phyla (132).

Carbon monoxide, present at ∼90 ppbv in the atmosphere (133), is also aerobically respired by soil microbial communities. Physiological studies have shown that the enzyme responsible for this process, carbon monoxide dehydrogenase (134), is also induced during carbon limitation and enhances survival during starvation (124, 135–138). At least four microbial phyla can scavenge atmospheric CO (135), namely, *Actinobacteria* (135, 139), *Proteobacteria* (140, 141), *Chloroflexi* (124, 142), and *Euryarchaeota* (143, 144). Moreover, a recent genomic survey identified putative CO dehydrogenase genes in 16 microbial phyla, encompassing most of the dominant taxa detected in soils (135).

Increasing evidence suggests that oxidation of atmospheric H_2_ and CO is a feasible continuous-energy-harvesting strategy for microorganisms living in desert ecosystems. Indeed, the genetic determinants for these reactions are consistently detected in desert surveys. Analysis of metagenomic and metatranscriptomic sequence data from the Colorado Desert and Tarim Basin revealed that the genes encoding these enzymes are both abundant and expressed by the soil microbial communities (135). Metagenome-assembled genomes of bacteria with trace gas oxidation potentials, including *Pseudonocardia* from the high-elevation Atacama Desert (145) and “*Candidatus* Dormibacteraeota,” “*Candidatus* Eremiobacteraeota,” *Actinobacteria*, *Chloroflexi*, and *Verrucomicrobia* from Robinson Ridge, Antarctica (132), have been recovered from hyper-arid mineral soils. Experimentally, the rapid consumption of H_2_ and CO to subatmospheric concentrations was demonstrated in microcosm experiments using Antarctic soils (132). In addition, calculations of theoretical energy yield from trace gas oxidation suggest that this process is sufficient to support the maintenance energy requirement of soil microbial communities (132, 146). Trace gas oxidation may explain why *Actinobacteria* is the dominant bacterial phylum in desert soils. The relative abundance of this phylum increases with aridity (147, 148), and it typically accounts for 30 to 80% of the microbial community in hyper-arid sites (7, 13, 132, 145, 147, 149) (Fig. 1). Concomitantly, genome-mining data suggest that group 1h [NiFe] hydrogenase genes (123) and carbon monoxide dehydrogenase genes (135) are universally distributed within this phylum. Importantly, CO and H_2_ oxidation can remain active at low water potentials, with the slow uptake of atmospheric CO detectable at water potentials between −41 MPa and −117 MPa (143, 150), comparable to values in hyper-arid desert soils.

Methane, present at 1.9 ppmv, is the most abundant reduced gas in the troposphere (151). Unlike atmospheric H_2_ and CO oxidation, it is likely that atmospheric CH_4_ oxidation has a limited role in supporting microbial persistence in desert environments. This compound is oxidized to methanol by particulate and soluble methane monooxygenases, which is further oxidized for energy production or carbon assimilation (152). To date, taxa with the ability to oxidize CH_4_ at atmospheric concentrations are exclusively found in specific lineages of the *Alphaproteobacteria* and *Gammaproteobacteria* (153). In desert soil ecosystems, atmospheric CH_4_ oxidation has been reported (154–157). However, the observation of detectable methane oxidation and methane monooxygenase genes in different samples is highly sporadic, especially in more arid soils (145, 155, 156, 158). Moreover, the activity of CH_4_ oxidation and the abundance of methanotrophs appear to decline dramatically at low water content (117, 150, 159). However, it is known that some methylotrophs are in high relative abundance in some desert soils; an example is Methylobacterium radiotolerans, which dominates the microbial communities at depths below 5 meters in the Playa of the Atacama Desert soils (94).

## CONCLUSIONS

Recent advances in “omics” techniques, in combination with pure culture studies and sensitive biogeochemical measurements, have enabled a rapid expansion of knowledge of the diversity and function of organisms living in water-scarce environments. It is now acknowledged that surprisingly diverse microbial communities survive in even the most arid and oligotrophic soils, such as the Antarctic cold deserts and the Atacama Desert. In the absence of macrophytic phototrophs, these microorganisms are the predominant contributors to primary productivity and biogeochemical activities. However, our understanding of how these organisms survive during long periods of water deficiency and how biodiversity in arid soil environments is maintained and shaped remains incomplete. Here, we have presented two strategies for microbial survival in arid ecosystems that can sustain dormancy: the energy reserve hypothesis and the continual-energy-harvesting hypothesis. The strategies are certainly not mutually exclusive, but their degree of relative importance is likely to vary according to the severity of different environmental parameters, such as light availability, oligotrophy, and water availability (Fig. 2). A deeper understanding of these mechanisms is likely to contribute substantially to our capacity to predict how ecosystems, as well as the services that they provide, are affected by the projected global desertification.
